# Sainfoin (*Onobrychis* spp.) crop ontology: supporting germplasm characterization and international research collaborations

**DOI:** 10.3389/fpls.2023.1177406

**Published:** 2023-05-15

**Authors:** Ebrar Karabulut, Kübra Erkoç, Murat Acı, Mahmut Aydın, Spencer Barriball, Jackson Braley, Eric Cassetta, Evan B. Craine, Luis Diaz-Garcia, Jenna Hershberger, Bo Meyering, Allison J. Miller, Matthew J. Rubin, Omar Tesdell, Brandon Schlautman, Muhammet Şakiroğlu

**Affiliations:** ^1^ Bioengineering Department, Adana Alparslan Türkeş Science and Technology University, Adana, Türkiye; ^2^ The Land Institute, Salina, KS, United States; ^3^ Department of Computer Engineering, Kafkas University, Kars, Türkiye; ^4^ Donald Danforth Plant Science Center, St. Louis, MO, United States; ^5^ Department of Viticulture and Enology, University of California Davis, Davis, CA, United States; ^6^ Plant and Environmental Sciences Department, Clemson University, Clemson, SC, United States; ^7^ Department. of Biology, Saint Louis University, St. Louis, MO, United States; ^8^ Department of Geography, Birzeit University, Birzeit, West Bank, Palestine

**Keywords:** sainfoin, *Onobrychis* spp., crop ontology, perennial grain, pulse, forage

## Abstract

Sainfoin (*Onobrychis* spp.) is a perennial forage legume that is also attracting attention as a perennial pulse with potential for human consumption. The dual use of sainfoin underpins diverse research and breeding programs focused on improving sainfoin lines for forage and pulses, which is driving the generation of complex datasets describing high dimensional phenotypes in the post-omics era. To ensure that multiple user groups, for example, breeders selecting for forage and those selecting for edible seed, can utilize these rich datasets, it is necessary to develop common ontologies and accessible ontology platforms. One such platform, Crop Ontology, was created in 2008 by the Consortium of International Agricultural Research Centers (CGIAR) to host crop-specific trait ontologies that support standardized plant breeding databases. In the present study, we describe the sainfoin crop ontology (CO). An in-depth literature review was performed to develop a comprehensive list of traits measured and reported in sainfoin. Because the same traits can be measured in different ways, ultimately, a set of 98 variables (variable = plant trait + method of measurement + scale of measurement) used to describe variation in sainfoin were identified. Variables were formatted and standardized based on guidelines provided here for inclusion in the sainfoin CO. The 98 variables contained a total of 82 traits from four trait classes of which 24 were agronomic, 31 were morphological, 19 were seed and forage quality related, and 8 were phenological. In addition to the developed variables, we have provided a roadmap for developing and submission of new traits to the sainfoin CO.

## 1 Introduction

Sainfoin (*Onobrychis* spp. Fabaceae) has a long and rich history of cultivation across Asia, Europe, and North America where it is used to provide roughage for livestock and to maintain soil fertility (Frame et al., 1998). Sainfoin has been used as a perennial forage legume and in crop rotation regimes with major grains such as wheat and barley (Hayot Carbonero et al., 2011). Sainfoin use has been centered in Turkey, the Balkans, and Central and Southern Europe (Bennett et al., 2001), but historical evidence suggests it was also grown in Palestine, Syria, and Lebanon in the late 1800s (Tristram, 1885). The introduction of synthetic fertilizer-based production schemes led to a reduction in sainfoin cultivation in these regions (Hayot Carbonero et al., 2011), and a concomitant decline in research and breeding efforts. Recently, as concerns about synthetic fertilizers have grown, attention has refocused on the crucial role of legumes in agricultural systems, and as a result, interest in sainfoin has been revived (Şakiroğlu, 2021; Craine et al., 2023).

Agriculture and plant breeding are undergoing a revolution in response to calls for the development of more diverse, sustainable, agricultural systems. A key part of this is plant breeding, the improvement of existing crops and development of new ones that provide agronomic products and critical ecosystem services. For example, there is emerging interest in domesticating sainfoin as a potential novel, sustainable food source - a perennial pulse - for human consumption (Butkutė et al., 2018a; Butkutė et al., 2018b; Schlautman et al., 2018). Evidence from nutritional analyses and animal feeding studies suggest sainfoin seeds could be suitable for human and animal consumption (Ditterline, 1973; Tarasenko et al., 2015; Baldinger et al., 2016; Wijekoon et al., 2021; Craine et al., 2023). Thus, recent breeding efforts to develop sainfoin for dual-purpose perennial grain and forage production by selecting phenotypes related to grain yield and grain quality have begun at The Land Institute (Salina, KS, USA), Adana Alparslan Türkeş Science and Technology University (Adana, Turkey), and elsewhere.

As plant breeding programs expand and evolve to meet current and future agricultural needs, it is necessary to adapt existing frameworks for cataloging plant information. In the post-omics era (e.g. genomics, transcriptomics, proteomics, metabolomics, hormonomics, ionomics, and large-scale automated phenomics) the amount and complexity of data collected, stored, and shared within and among breeding and agriculture research programs has reached an all-time high (Langridge and Fleury, 2011; Leonelli et al., 2017; Li and Yan, 2020; Van Tassel et al., 2022). These post-omics era technologies promise to generate more data at lower costs than ever before, which could accelerate genetic gains in underutilized crops or even rapidly domesticate new ones. However, leveraging these technologies and large datasets when collaborating is only possible with available infrastructure to robustly store and access data. As such, there is a need for new frameworks that enable breeders to efficiently share and communicate about the multi-dimensional plant phenotypes characterized in their programs, for different breeding goals, in a broader diversity of current and emerging crop species like sainfoin.

As early as the 1990s, the need for designing databases serving multiple users with a robust set of common ontologies was recognized (Wieczorek et al., 2012). Numerous ontologies and ontology platforms have since been created to support and standardize data sharing within and among research fields such as Darwin Core (https://dwc.tdwg.org) as a standard for biodiversity data and the Planteome platform (https://www.planteome.org) and related Plant Ontology (PO), Plant Trait Ontology (TO), and Plant Experimental Conditions Ontology (PECO) frameworks, which provide a base for ontologies for plant and species-specific traits related to plant development, anatomy, physiology in the context of genomics data (Jaiswal et al., 2005; Pujar et al., 2006; Ilic et al., 2007; Avraham et al., 2008; Arnaud et al., 2012; Cooper et al., 2013; Cooper and Jaiswal, 2016; Cooper et al., 2018).

Crop Ontology (CO, https://cropontology.org, Matteis et al., 2013)) was created in 2008 by the CGIAR to provide a framework and common language to catalog crop-specific trait data, allowing traits to be easily interpretable and interoperable for further aggregation, analysis, and multidisciplinary communication (Gruber, 2009). CO currently supports the standardization of plant breeding databases such as the Integrated Breeding Platform’s BMS (IBP; https://www.integratedbreeding.net/), the Boyce Thompson Institute’s Breedbase (https://breedbase.org/, (Morales et al., 2022), and others (Arnaud et al., 2020) which allow the creation and management of annotated trial data (Crop Ontology 2022). The Minimum Information About a Plant Phenotype Experiment (MIAPPE https://www.miappe.org/; (Ćwiek-Kupczyńska et al., 2016; Papoutsoglou et al., 2020) and the Breeding Application Programming Interface (BrAPI; https://brapi.org/; (Selby et al., 2019) have both adopted the CO format, demonstrating the widespread acceptance and utility of the standard (Arnaud et al., 2020). The CO Application Programming Interface (API) is used by third-party websites and databases like the EMBL-EBI Ontology Lookup Service that replicates CO and provides term search access through its own portal. Agroportal, the registry of ontologies in agriculture and related domains, regularly synchronizes their files with CO.

Several different COs have since been developed and made accessible through the CO platform by research groups and crop specific consortiums for several commonly cultivated crops including apple, banana, cotton, corn, common bean, potato, rice, and wheat. We expect that the broader impacts made possible through international and transdisciplinary collaboration and germplasm characterization in sainfoin can be magnified through early efforts of a consortium of researchers, hereafter referred to as the “Sainfoin Consortium.” In this current work, we describe the efforts of the Sainfoin Consortium to standardize the nomenclature and data storage systems used for sainfoin research to create a sainfoin CO - the first CO developed for a perennial forage legume and grain crop. We also include a roadmap for further expansion of the sainfoin CO through a set of guidelines for the development and suggestion of new traits.

## 2 The sainfoin ontology framework

### 2.1 Ontology construction methods

We used the Crop Ontology framework guidelines (version 2.1; Pietragalla et al., 2022) and trait dictionary template to build the first version of a sainfoin crop ontology. The online database software, Airtable, was used to create the first version of the ontology as it incorporates relational data structures that can be used to easily link ontology terms across tables in the database. Figures and entity relation diagrams were constructed in DrawIO (https://github.com/jgraph/drawio). The Crop Ontology CGIAR advisory board assigned the crop code CO_369 to sainfoin, which is prepended to Variable terms in the final sainfoin ontology. The sainfoin crop ontology is available to the public, can be browsed on the Crop Ontology website (https://cropontology.org/term/CO_369:ROOT), resides in a dedicated GitHub repository (https://github.com/Planteome/CO_369-sainfoin-traits), and is maintained by a group of community curators from the Sainfoin Consortium.

### 2.2 Term types and structure

The CO phenotype annotation model is based on three fundamental CO term types: Trait, Method, and Scale. These three fundamental types are then used in conjunction to construct a fourth term type, Variable, which formalizes how a given trait is collected. Each CO term in the sainfoin ontology was assigned a persistent unique identifier (PUID), which is composed of the sainfoin CO crop code and a seven-digit number in the form {CO crop code}:{#######}, e.g., CO_369:0000001. While the format of the ID system is constrained as shown above, the CO guidelines do not mandate a particular system for assigning PUIDs to Variables, Traits, Methods, or Scales within an individual ontology. To maintain an incremental, identifiable PUID system, we added constraints for each term type (Variable, Trait, Method, and Scale) shown in **Table 1**.

**Table 1:** Term type ID series composition and creation.

Term Type	Term PUID Base Series	Term PUID Constraints
Variable	CO_369:0000000	1-999
Trait	CO_369:0001000	1001-1999
Method	CO_369:0002000	2001-2999
Scale	CO_369:0003000	3001-3999

The Sainfoin Crop Ontology V1 increments each term type from the base series within the constraints of each term type. This allows for the development of 999 unique terms for each term type while enforcing strict identifiability for each term type.

In addition to PUIDs, all terms in the ontology were given human-readable names and abbreviations that can be used in trait selection used in data collection. We avoided using acronyms within CO term names except where the term name would become unwieldy without its use or when the acronym is more widely known than the actual phrase which the acronym represents (e.g., ‘SPAD’ for soil plant analysis development). Term names were constrained to standard ASCII characters, and aside from acronyms, only the first letter of the first word in each name is capitalized.

### 2.3 Trait composition framework

A Trait in the Sainfoin CO is a subcomponent of a variable that defines what is observed. Traits are composed of a meaningful, two- to four-word phrase consisting of an Entity, the observed part of a plant, and an Attribute, a feature of an entity, in the form {Entity} {Attribute} such as {Plant} {height}. Traits are then assigned to one of the nine Trait classes specified in the CO guidelines for organizational purposes when viewing the ontology (**Table 2**). When possible, Entities and Attributes in the sainfoin ontology were cross referenced with terms in existing relevant ontologies such as PO, the Agronomy Ontology (AGRO, Aubert et al., 2017), The Environment Ontology (ENVO, Buttigieg et al., 2016) and The Phenotype And Trait Ontology (PATO, Gkoutos) to standardize term vocabulary across other ontology frameworks.

**Table 2:** List of trait classes, descriptions, and corresponding frequencies in the Sainfoin Crop Ontology.

Trait class	Absolute frequency	Relative frequency	Class Description
Agronomic	24	0.29	All main traits contributing to yield and related to the agronomic performance of plants.
Morphological	31	0.38	All traits related to anatomical and morphological structure of the plant, its organs, and tissues.
Quality	19	0.23	All traits related to key characteristics that influence end-use quality of crop/plant products and sub-products.
Phenological	8	0.10	All traits related to growth/developmental stages and periods of crop/plants.
Abiotic Stress	0	0.0	All traits related to stress caused by non-living stressors.
Biochemical	0	0.0	All traits related to chemical components of a plant entity.
Biotic Stress	0	0.0	All traits related to stress caused by living stressors.
Fertility	0	0.0	Traits specifically related to fertility aspects of importance to breeding.
Physiological	0	0.0	All traits related to the functioning of the crop/plant and its response/adaptation to the environment.
**Total**	82	1.00	

Extensive examination of ontologies from other species revealed inconsistent approaches for assigning Entities and Attributes, especially those that can have multiple states. This multiple state problem is common when a treatment or processing step is applied (e.g., drying, boiling, milling). Using the example of the Entity {Forage} and the attribute {mass}, which can be measured either in a fresh or dried state. The CO guidelines specify two distinct approaches for assigning the state “dry”, but each has its own challenge.

1. The “dry” state is assigned to the Entity {Dry forage} rather than the Attribute{mass}.

a. This approach creates a hierarchy of entities with multiple states rather than treating entities as a single observed part of a plant.

2. The “dry” state is not assigned to either the Entity {Forage} or Attribute {mass} but is instead included in the Method describing how Forage mass was measured either fresh or dry.

b. This approach results in multiple traits with the same name, which can create downstream challenges for users in selecting the proper Variable in tools such as Field Book (Rife and Poland, 2014) or Gridscore (Raubach et al., 2022). This is especially important in the sainfoin ontology where only the Method class abbreviation is included in the Variable name.

We chose a third approach, which was to assign the state to the attribute instead of the entity or method. In this fashion, any traits with state(s) would be constructed as {Entity} {(state) attribute} as in Forage dry mass and Forage fresh mass. With this format, we can have Methods that simply describe the Method for a given Trait(s) without having to form specific Methods for each Trait that only differ in the state of a Trait.

Finally, when choosing Attribute words, we opted for words that were specific enough to be contextually correct, but general enough that they could be used in more than one context. For example, “mass” was used instead of “weight”, and “mass” was chosen over “biomass” since the latter adds no further meaning when the context is already scoped to biological organisms.

### 2.4 Method composition framework

Methods are the component of a Variable that describes how an observation is made. The framework we followed for composing Methods was based on the outline specified in the CO guidelines, with some modifications that add clarity to the procedures and allow for some flexibility between different breeding programs’ goals. Our modifications fall into three main categories: Method name, Method description, and Method abbreviation.

First, we constrain the Method name to be a succinct, human-readable name appended with the Method class abbreviation. In most cases this should be the trait name followed by the Method class the Method belongs to following the format of {Trait} {Method class}. For example, ‘Leaflet SPAD measurement’ tells us this Method describes how to measure SPAD values of a leaflet. However, exceptions were made when general Methods could be assigned to multiple traits, as in the case of ‘Object equivalent diameter measurement’, which describes a simple image processing technique that could be applied to many different objects in an image. Methods were categorized into one of the seven classes (measurement, counting, estimation, computation, prediction, description, or classification) defined in the CO Guidelines.

**Figure 1:**
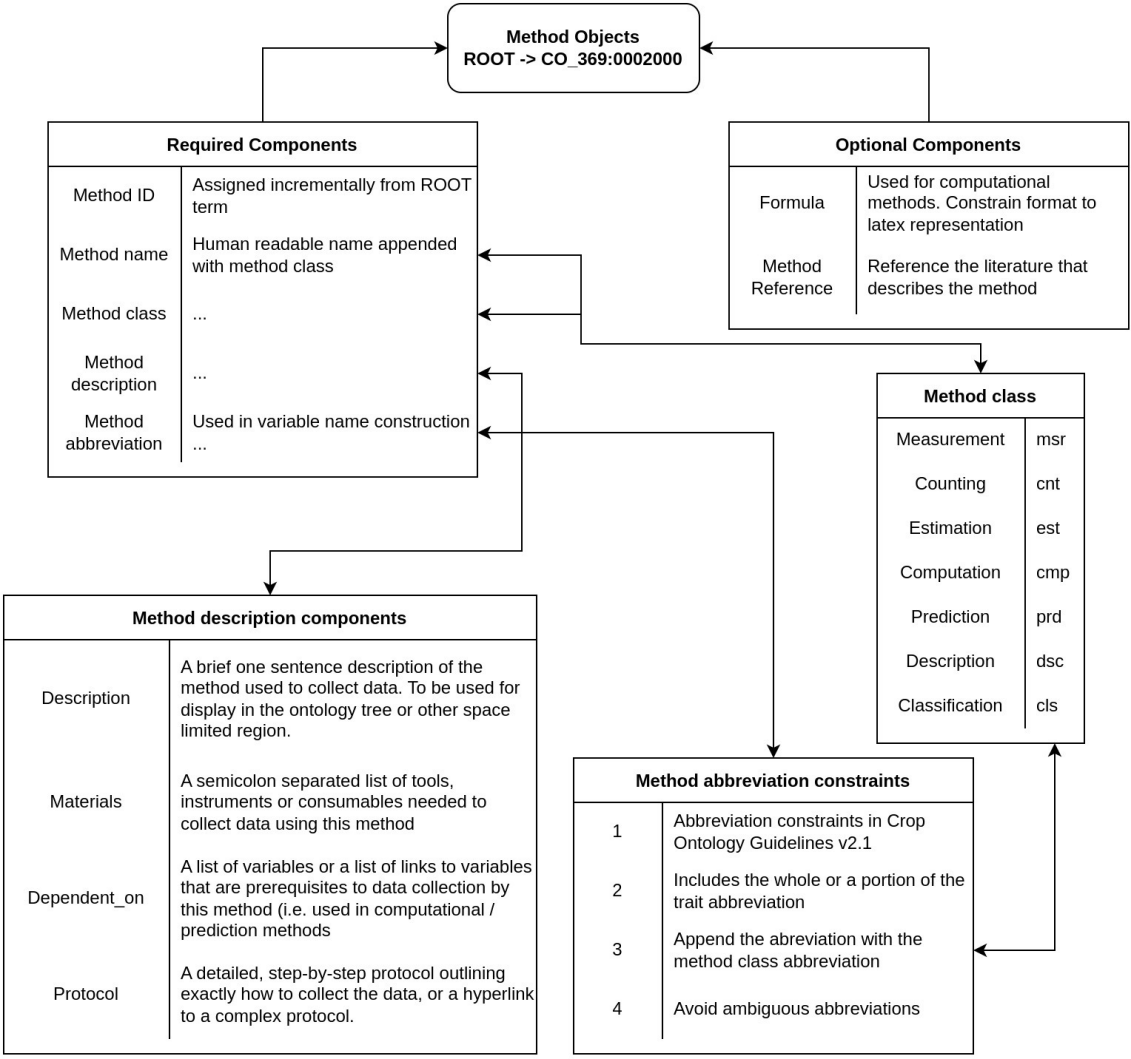
Schematic representation of the Method development for the Sainfoin Crop Ontology.

Second, the Method description should be structured according to the format shown in **Figure 1**. While the Method description is allowed to be a free text field in the CO guidelines, we constrained our descriptions to include at minimum the following structured information: ***Description***: A brief, one sentence description of the Method; ***Materials***: A semicolon separated list of materials or supplies needed for the Method; ***Dependent_on***: A semicolon separated list of Variables that the Method uses for computational purposes or for normalization. Variables can be specified either by the variable label (Raceme length msr [cm]), ID (CO_369:0000061), or name (RaL_RaLmsr_cm); ***Protocol***: A detailed, ordered list of steps in the protocol needed to complete the Method. When it is infeasible to write out a lengthy protocol, it is indicated by writing “See full protocol in {publication title, author, year}”.

Many existing CO Method descriptions either present no added information beyond that which is already specified in the Method name or present methodology too vague to be followed without error. We developed our Method descriptions to be agnostic regarding the sampling procedure so that the employment of a given Method can be adapted to the end user’s specific experimental design. This modification is not entirely in line with the CO guidelines which state that the sampling should specify whether the observation is collected on a single plant or an aggregation from multiple plants (an experimental group or plot). However, this poses an issue with the scope of sample sizes (e.g., number of samples aggregated) commonly seen in breeding or agronomic trials. Specifically, a trait collected in greenhouse studies using single plant reps should, according to this guideline, have a separate method from the same trait collected in a field trial with collections of plants in a plot or sward. This leads to, at most, doubling the number of variables in the ontology. Furthermore, while the guidelines state that the experimental protocol should be distinguished from the observational protocol, the authors realize that the sampling protocol is inevitably linked to the experimental design and informs the observation procedure. Various research groups have their preferred sampling methods, dependent on goals, scale of projects, funding, etc.; therefore, we leave the specific sampling procedure up to the individual researchers and only dictate that the trait value is represented *as-is* for data collected on one observational unit, or the mean/aggregated value for a collection of observational units.

Finally, we constrain the abbreviation of the Method used in Variable name construction to include the Trait abbreviation, or if not possible, at least a portion of the Trait abbreviation. Ambiguous abbreviations such as ‘PH’ for ‘Plant height’ or ‘pH’ were avoided. Additionally, the standard abbreviation of the Method class is appended at the end: msr, cnt, est, cmp, prd, dsc, or cls (See **Supplemental Table 3**). This is to avoid confusion about what type of methodology is being employed in a Variable.

New Methods are to be developed following the above guidelines as schematized in **Figure 1**. Briefly, there are five obligatory Method components: Method ID, Method name, Method class, Method description, and Method abbreviation. The Method ID is assigned automatically and incrementally. The Method class must be selected among the seven different Method classes (**Figure 1**).

### 2.5 Scale composition framework

Scales are the component of a variable that describes how the observation is expressed. Scales were composed of units in the International System of Units (SI) with their associated official abbreviations. Scales were cross referenced to the Units of measurement Ontology (Gkoutos et al., 2012) or other ontologies such as PO and AGRO when applicable. Units with a ‘μ’ prefix were included even though they are not a part of the ASCII standard character set, however, ‘u’ may be used in place of ‘μ’ when convenient. Scales were specified as either a Date, Duration, Nominal, Numerical, Ordinal, Text, or Code Scale class.

### 2.6 Variable composition framework

**Figure 2:**
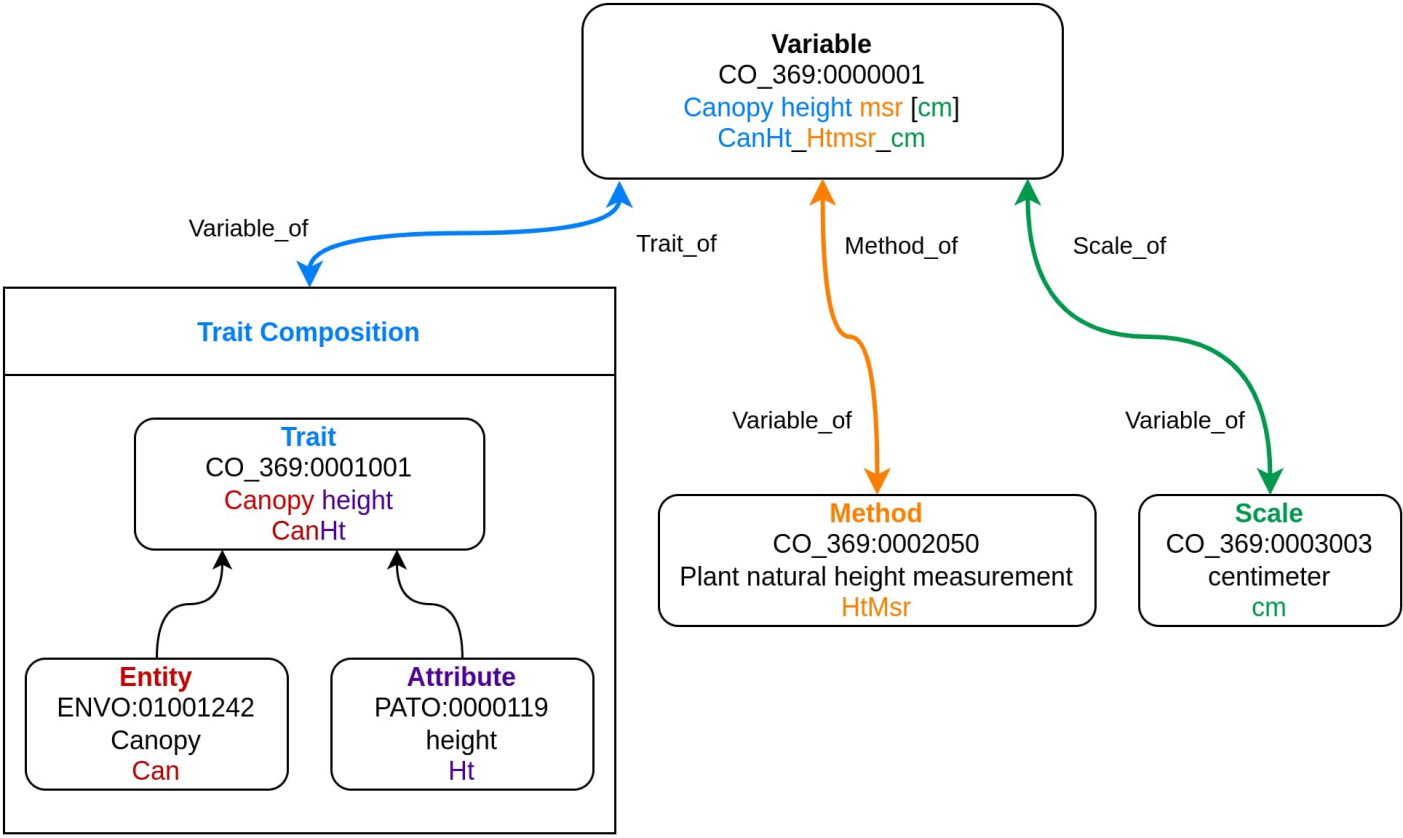
Schematic representation of Variable term composition in the Sainfoin Crop Ontology. In this example, the Variable ‘Canopy height msr [cm]’ is used to demonstrate how complex ontology terms are constructed of simpler terms and how the term identification number system functions. For each term type, (Variable, Trait, Method, and Scale) the PUID, term label and abbreviations are listed in order.

Variables are the breeder’s or agronomist’s observations or measurements. The CO model defines a Variable as a unique combination of a Trait, Method, and Scale (Variable = Trait + Method + Scale), which allows for standardized data collection, storage, and sharing. Variable labels are human readable: they are composed of a Trait name followed by an associated Method class abbreviation, and a Scale enclosed in square brackets (e.g., Seed length msr [mm]); and used in scientific discussions and publications. Variable abbreviations, also referred to as names in the CO guidelines, are composed of {Trait abbreviation}_{Method abbreviation}_{Scale abbreviation} with no further modifications, (e.g., ‘SdL_Lmsr_mm’); and used as unique IDs in databases, analyses, and phenotyping applications. A detailed schematic of Variable composition is shown in **Figure 2**.

## 3 Populating the sainfoin ontology

### 3.1 Gathering a broad target list and shortlist of most used traits

Despite limited interest in sainfoin in the research community, a wealth of research targeting various aspects of the crop has been published in different languages. In addition to forage yield and quality traits, adaptability, resistance to biotic and abiotic stress factors, biochemical, physiological, morphological and phenological traits have been investigated. Research targeting cytogenetic, morphological, and molecular aspects along with taxonomic status of sainfoin and allied taxa have also been investigated and reported (Aktoklu, 1995; Hayot Carbonero et al., 2011; Kempf et al., 2017; Şakiroğlu, 2021).

We performed an in-depth literature review in the languages accessible to our consortium (English and Turkish) to develop a comprehensive list of Variables that have been previously measured and reported in sainfoin. The process of determining which of these variables to include, and the specific Trait, Method, and Scale terms to use, required many discussions, compromises, and decisions that spanned multiple months. The terms we included reflect the traits, methods, and scales currently being used in breeding and research programs led by the authors of this manuscript. In some instances, multiple variables are included for the same trait reflecting the differences in methods or scales used within the consortium. We selected 76 sainfoin Variables to include in sainfoin CO v1, of which the majority are from the agronomic, morphological, quality, and phenological trait classes (**Table 2**). We observed many traits from other classes (e.g. abiotic stress and biotic stress) during our literature review, but did not incorporate them into Variables in the first version of the sainfoin CO. We have included a list of 79 Traits that could be added to the sainfoin CO in the future as our community’s expertise or interest in these other trait classes expands (**Supplementary Table 1**).

### 3.2 Adding quality traits relevant to sainfoin as a perennial grain crop

Developing sainfoin as a new perennial pulse will require measuring new traits that are typically relevant only to cereal and grain legume crops. We reviewed the ontologies for several grain crops, including wheat, barley, oat, and common bean, that are available on the CO platform, and we compiled a list of 258 Variables from multiple trait classes that have not been previously measured in sainfoin but might be applicable to sainfoin breeding as a perennial pulse crop (**Supplemental Table 2**). While cereals provide a broad frame for grain related traits, common bean traits are particularly relevant, serving as a source of legume specific traits.

A comprehensive understanding of the chemical composition and nutritional quality of sainfoin seeds is needed to determine the safety of this new food source and the nutritional value of sainfoin as a novel pulse crop. Some of these quality traits, such as crude protein, dietary fiber, and phytic acid content, were recently measured in sainfoin for the first time (Craine et al., 2023). Compared to other pulses, depodded sainfoin seeds have higher protein content, as reported by Baldinger et al. (2016) (38.8%), Woodman and Evans (1947) (36.6%), Craine et al. (2023) (38.78%), and Ditterline (1973) (36.0%), and comparable iron and zinc content, as reported by Craine et al. (2023) (Fe, 56.25 - 74.24 ppm; Zn, 54.78 - 79.05 ppm), each of which plays a vital role in human health.

Of the many potential “grain” related traits, we chose to create only 8 Variables related to seed quality in the initial sainfoin CO, which were recently profiled in a study evaluating sainfoin seed attributes (Craine et al., 2023). Creating Variables and appropriate terms for these eight seed quality Traits, namely, protein, crude fat, carbohydrates, total starch, dietary fiber, iron, zinc, and phytic acid content, was simplified by their common use across many crop species (**Table 3**). These quality Traits are measured using methods approved by the Association of Official Agricultural Chemists (AOAC) and/or American Association of Cereal Chemists (AACC), and the appropriate AOAC and AACC method codes are referenced in the related Method description in the sainfoin CO.

**Table 3:** Variables from Craine et al. (2023) that are included in the Sainfoin Crop Ontology V1.

Variable	Trait		Method		Scale	
	Name	Class	Protocol	Class	Name	Class
Seed protein content msr [DMB*]	Seed crude protein	Quality	AACC 46-30.01	Measurement	g/100g	Numerical
Seed crude fat content msr [DMB]	Seed crude fat content	Quality	AACC 30-25.01 (ETHER EXTRACTION)	Measurement	g/100g	Numerical
Seed carbohydrate content cmp [DMB]	Seed carbohydrates	Quality	100 - (ASH + MOISTURE + FAT + PROTEİN)	Computation	g/100g	Numerical
Seed total starch content msr [DMB]	Seed total starch content	Quality	AOAC 996.11	Measurement	g/100g	Numerical
Seed dietary fiber content msr [DMB]	Seed dietary fiber content	Quality	AACC 32-07.01/AOAC 991.43	Measurement	g/100g	Numerical
Seed iron content msr [DMB]	Seed iron content	Quality	AACC 40-70.01	Measurement	ppm	Numerical
Seed zinc content msr [DMB]	Seed zinc content	Quality	AACC 40-70.01	Measurement	ppm	Numerical
Seed phytic acid content msr [DMB]	Seed phytic acid content	Quality	HPLC RI	Measurement	mg	Numerical

*DMB, Dry matter basis.

## 4 Future perspectives

### 4.1 Guidelines for contributing to the sainfoin ontology

The sainfoin CO represents a necessary step towards making sainfoin research accessible and discernable to an international community of researchers. However, the sainfoin CO is far from complete. Sainfoin has tolerance to various biotic and abiotic stresses, and traits related to sainfoin drought and salinity tolerance would be of immense importance in agriculture (Heinrichs, 1972; Juan, 1982; Morrill et al., 1998; Meyer and Badaruddin, 2001; Irani et al., 2015; Kölliker et al., 2017). We encourage researchers with expertise and experience in areas not represented in the current sainfoin CO to contribute to expanding its scope and utility in the future.

Researchers can suggest and submit new sainfoin ontology terms (I.e., Variables, Traits, Methods, or Scales) through https://trait-requests.planteome.org/or a GitHub issues template form. Extant terms can be updated with sufficient rationale, or term synonyms can be suggested where two competing names are commonly used to describe that term. Any new terms should meet the baseline guidelines laid forth in the official CO Guidelines v2.1, and conform to the additional requirements and constraints set forth above. Such a system will aid in constructing a more helpful ontology. Two curators (English and Turkish native speakers) are actively maintaining and improving the sainfoin CO. The curators are notified upon any new term submission, and follow-up discussions about the term are handled through GitHub issues.

### 4.2 The roles of crop ontologies in developing new sustainable crops and cropping systems

In this manuscript, we share our experiences in building the sainfoin CO in hopes that we can continue improving research infrastructure for the international sainfoin community and to provide a template for future crop ontology development for other perennial grains, forages or minor pulse crops. There is global recognition for the growing need for agroecosystem sustainability and resilience to climate change (FAO (Food and Nations), A. O. of the United Nations, 2018; Tittonell, 2020). Sainfoin, as both a perennial pulse and perennial forage, has the potential to contribute towards these goals internationally; however, many other new and underutilized crops will be needed in various contexts. We expect that data infrastructure, such as the sainfoin CO presented herein, combined with technology to collect multi-dimensional data at scales and rates higher than ever before, will allow researchers from multiple languages and research disciplines to collaborate effectively to make rapid progress towards domesticating new perennial grains, developing new sustainable cropping systems, and preparing agriculture internationally for climate challenges in the future.

## Data availability statement

The datasets presented in this study can be found in online repositories. The names of the repository/repositories and accession number(s) can be found below: https://cropontology.org/term/CO_369:ROOT https://github.com/Planteome/CO_369-sainfoin-traits.

## Author contributions

Conceptualization: BS, MS. Data curation - EK, KE, ECr, MAc, MAy, SB, BM, JB. Funding acquisition - BS, MS, AM. Methodology- BM, SB, JH, EK, KE, ECr, SB, LD, OT. Project administration - BS, MS, BM. Software - MAy, BM. Supervision - MS, BS. Visualization – BM. Writing – original draft - MS, BM, BS, KE, EK, ECr, ECa, MR. Writing – review & editing - All authors. All authors contributed to the article and approved the submitted version.

## Supplementary material

Supplementary Table 1 List of important Traits and Variables in sainfoin as determined by literature review.

Supplementary Table 2 Traits and variables from wheat, oat, barley, and common bean that might be relevant to sainfoin.

Supplementary Table 3 List of entities and descriptions in the ontology.
